# *IL-10* and *PRKDC* polymorphisms are associated with glioma patient survival

**DOI:** 10.18632/oncotarget.13028

**Published:** 2016-11-02

**Authors:** Mingjun Hu, Jieli Du, Lihong Cui, Tingqin Huang, Xiaoye Guo, Yonglin Zhao, Xudong Ma, Tianbo Jin, Gang Li, Jinning Song

**Affiliations:** ^1^ Department of Neurosurgery, First Affiliated Hospital of Medical College, Xi'an Jiaotong University, Xi'an 710061, China; ^2^ Department of Neurosurgery, Xi'an First Hospital, Xi'an 710002, China; ^3^ Inner Mongolia Medical University, Hohhot, Inner Mongolia, 010050, China; ^4^ Department of Orthopedics and Traumatology, The 2nd Affiliated Hospital of Inner Mongolia University, Hohhot, Inner Mongolia, 010030, China; ^5^ Department of Neurology, Shangluo Central Hospital, Shangluo 726000, China; ^6^ Key Laboratory of Resource Biology and Biotechnology in Western China, Northwest University, Ministry of Education, School of Life Sciences, Northwest University, Xi'an 710069, China; ^7^ Department of Neurosurgery, Tangdu Hospital, The Fourth Military Medical University, Xi'an 710038, China

**Keywords:** IL-10, PRKDC, biomarker, glioma, polymorphism

## Abstract

*Interleukin-10* (*IL-10*) and DNA repair gene *PRKDC* mutations are implicated in the development of multiple human cancers, including glioma. We investigated associations between *IL-10* and *PRKDC* gene polymorphisms and prognosis in low- and high-grade glioma patients. We analyzed the associations of one *IL-10* and one *PRKDC* single nucleotide polymorphism with patient clinical factors in 481 glioma patients using Cox proportional hazard models and Kaplan-Meier curves. We also assessed associations between patient clinical characteristics and prognosis. Our data showed that the extent of tumor resection (gross-total resection) and application of chemotherapy were associated with improved patient outcomes in all glioma cases. Additionally, univariate (Log-rank *p* = 0.019) and multivariate Cox regression analyses (*p* = 0.022) showed that the *IL-10* rs1800871 C/T genotype correlates with improved overall survival in cases of low-grade glioma, whereas the *PRKDC* rs7003908 C/C genotype correlated with reduced overall and progression-free survival in high-grade glioma patients in univariate (Log-rank *p* = 0.000 and *p* = 0.000, respectively) and multivariate Cox regression analyses (*p* = 0.001; *p* = 0.002, respectively). These results suggest that *IL-10* rs1800871 and *PRKDC* rs7003908 may be useful biomarkers for predicting glioma patient outcome. Further functional studies are needed to evaluate the mechanisms by which these polymorphisms affect glioma progression.

## INTRODUCTION

Glioma is a general term used to describe any tumor that arises from the supportive (gluey) tissue of the brain. This tissue, called glia, helps to keep the neurons in place and functioning well. It is the most common and aggressive primary brain tumors, occurring most commonly in adults and accounting for 40–50% of all brain tumors [1]. According to the 2007 World Health Organization (WHO) classification, gliomas are subclassified into four grades based on degree of aggressiveness: grade I (pilocytic astrocytomas), grade II (diffuse infiltrating low-grade gliomas), grade III (anaplastic gliomas), and grade IV (glioblastomas multiforme) [2]. Low-grade gliomas (grade I–II) are well differentiated, and generally have better prognoses than high-grade gliomas (grade III–IV) [2, 3]. Despite total or near-total tumor resection, and advancements in chemo-radiotherapy, patient outcomes remain dismal. Median survival times for patients with grade III and IV gliomas are only 39 and 30 weeks, respectively [4].

Several clinical factors, such as surgical method, radiotherapy, chemotherapy, patient age and gender, and tumor grade, size, range and location, can affect glioma patient prognosis. Most gliomas result from the combined action of environmental factors and inherited genetic variations [5]. Haque, *et al.* [6] suggested that glioblastoma arises from genetic and epigenetic alterations in normal astroglial cells, implying that genetic factors are primarily responsible for gliomagenesis.

Interleukin-10 (IL-10), an important anti-inflammatory cytokine, is secreted by various immune cells. IL-10 has pleiotropic effects on immunoregulation and inflammation, and can promote carcinogenesis [7]. Huettner, *et al.* [8] suggested that IL-10 promotes glioma progression by enhancing tumor cell invasion and providing an immunosuppressive environment. Additionally, *IL-10* polymorphisms are associated with glioblastoma [9].

PRKDC, also known as human X-ray repair cross-complementing group 7 (*XRCC7*), is located on chromosome 8q11. Several studies have associated *PRKDC* with poor prognosis in numerous tumor types, such as esophageal cancer [10], B-cell chronic lymphocytic leukemias [11], and colorectal cancer [12]. *PRKDC* gene polymorphisms are also correlated with glioma risk [13, 14].

Although previous association studies linked *IL-10* and *PRKDC* genetic polymorphisms with glioma risk, few focused on the effects of these alterations on glioma patient prognosis. In the present study, we selected one single nucleotide polymorphism (SNP) in *IL-10* [9] and one in *PRKDC* [14], each of which was reportedly associated with glioma risk. We evaluated the associations between these two SNPs and low- and high-grade glioma patient prognosis in a Chinese population.

## RESULTS

### Patient clinical data

A total of 481 glioma patients were analyzed in this study, including 300 astrocytomas, 70 oligodendrocytomas, 43 glioblastomas, 31 ependymomas, 14 oligodendrogliomas, and 23 other types of gliomas. Detailed clinical data for glioma patients are summarized in Table 1. Patients included 264 (54.9%) men and 217 (45.1%) women, with 208 patients < 40 years of age and 273 ≥ 40 years of age. A total of 35 (7.3%) patients were classified as WHO grade I, 259 (53.8%) as WHO grade II, 124 (25.8%) as WHO grade III and 63 (13.1%) as WHO grade IV. 328 (68.2%) patients received gross-total tumor surgical resections and 153 (31.3%) received near-total or sub-total resections. Gamma knife radiotherapy was administered to 315 (65.5%) patients and conformal radiation therapy was administered to 122 (25.4%). Finally, 198 (41.1%) patients received chemotherapy. 438 (91.1%) patients had died at the last follow-up and 453 (94.2%) progressed during follow-up.

**Table 1:** Characteristics of glioma patients

Variable	Classification	Glioma		I-II		III-IV	
		No.of Patients	Percent%	No.of Patients	Percent%	No.of Patients	Percent%
Gender	Male	264	54.9	157	53.4	107	57.2
	Female	217	45.1	137	46.6	80	42.8
Age(years)	< 40	208	43.2	144	49.0	64	34.2
	≥ 40	273	56.8	150	51.0	123	65.8
WHO grade	WHO I	35	7.3	35	11.9		
	WHO II	259	53.8	259	88.1		
	WHO III	124	25.8			124	66.3
	WHO IV	63	13.1			63	33.7
Extent of resection	GTR	328	68.2	197	67.0	131	70.1
	STR or NTR	153	31.8	97	33.0	56	29.9
Radiotherapy	GK	315	65.5	197	67.0	118	63.1
	CRT	122	25.4	73	24.8	49	26.2
	No	44	9.1	24	8.2	20	10.7
Chemotherapy	Platinum	101	21.0	63	21.4	38	20.3
	Nimustine	60	12.5	35	11.9	25	13.4
	Temozolomide	37	7.7	26	8.8	11	5.9
	No	283	58.8	170	57.8	113	60.4
Survival condition	Survival	26	5.4	17	5.8	9	4.8
	Lost	17	3.5	14	4.8	3	1.6
	Death	438	91.1	263	89.5	175	93.6
Progress	Yes	453	94.2	275	93.5	178	95.2
	No	24	5.0	17	5.8	7	3.7
	Missing system	4	0.8	2	0.7	2	1.1

WHO: World Health Organization; GTR: Gross-total resection; NTR: Near-total resection; STR: Sub-total resection; GK: Gamma knife; CRT: Conformal radiation therapy.

### Univariate analysis

In a univariate analysis of clinical factors, including patient gender and age, extent of tumor resection, and radiotherapy and chemotherapy regimen, we found that the median overall survival (OS) of WHO grade I–II glioma patients (12 months; 3-year survival rate = 9.0%) was longer than that of WHO grade III–IV patients (10 months; 3-year survival rate = 5.5%) (Log-rank *p* = 0.039) (Tables 2–4). The extent of resection (gross-total resection) was a protective factor, as was chemotherapy, in all glioma patients. Overall, gross-total resection was associated with a 33.3 (Log-rank *p* = 0.000, HR = 0.667, 95% CI = 0.545–0.817, *p* = 0.000) and 36.5% (Log-rank *p* = 0.000, HR = 0.63.5, 95% CI = 0.519–0.777, *p* = 0.000) decrease in OS and PFS mortality hazards, respectively. Median OS and PFS were 11 and 8 months, respectively, with 10.2% and 6.0% 3-year survival rates. In low-grade glioma patients, gross-total resection was associated with a 32.3% (Log-rank *p* = 0.000, HR = 0.677, 95% CI = 0.522–0.878, *p* = 0.003) and 35.8% (Log-rank *p* = 0.000, HR = 64.2, 95% CI = 0.496–0.831, *p* = 0.001) decrease in OS and PFS mortality hazards, respectively. Median OS and PFS were 11 and 8 months, respectively, with 12.5% and 7.1% 3-year survival rates. Finaly, in high-grade glioma patients, gross-total resection was associated with a 37.6% (Log-rank *p* = 0.002, HR = 0.62.4, 95% CI = 0.451–0.864, *p* = 0.004) and 39.3% (Log-rank *p* = 0.001, HR = 60.7, 95% CI = 0.439–0.840, *p* = 0.003) decrease in OS and PFS mortality hazard, respectively. Median OS and PFS were 10 and 8 months, respectively, with 6.9% and 4.5% 3-year survival rates. Treatment with chemotherapy was also associated with an overall reduced risk of death as measured by increased patient OS (3-year survival rate = 14.2%, median survival = 12 months, Log-rank *p* = 0.000, HR = 0.643, 95% CI = 0.528–0.782, *p* = 0.000) and PFS (3-years survival rate = 11.6%, median survival = 8 months, Log-rank *p* = 0.000, HR = 0.731, 95% CI = 0.602–0.889, *p* = 0.002). This same trend was observed in OS (3-year survival rate = 16.2%, median survival = 12 months, Log-rank *p* = 0.000, HR = 0.665, 95% CI = 0.517–0.855, *p* = 0.001) and PFS (3-year survival rate = 13.3%, median survival = 8 months, Log-rank *p* = 0.004, HR = 0.722, 95% CI = 0.523–0.926, *p* = 0.010) of low-grade glioma patients, and in OS (3-year survival rate = 11.3%, median survival = 12 months, Log-rank *p* = 0.001, HR = 0.617, 95% CI = 0.450–0.846, *p* = 0.003) of high-grade patients. No significant correlations were identified between patient gender and age, WHO tumor grade, or radiotherapy and prognosis as measured by OS and PFS.

**Table 2:** Univariate analysis of the impact of clinical factors on glioma patient OS and PFS

Variable	Classification	No. of patients/events	OS					No. of patients/events	PFS				
			1/3-(year) SR (%)	MST	Log-rank *p*	HR (95%CI)	*p*		1/3-(year) SR (%)	MST	Log-rank *p*	HR (95%CI)	*p*
Gender	Male	264/241	30.3/7.0	11		1		262/248	17.6/4.9	8		1	
	Female	217/197	31.8/8.2	10	0.583	1.049 (0.869–1.267)	0.616	215/205	13.5/4.3	8	0.565	1.050 (0.872–1.263)	0.607
Age (years)	< 40	208/186	33.2/9.1	12		1		205/191	17.1/6.1	8		1	
	≥ 40	273/252	29.3/6.3	11	0.202	1.119/ (0.926–1.353)	0.244	272/262	14.7/3.5	8	0.135	1.136 (0.942–1.370)	0.182
WHO grade	I–II	294/263	33.0/9.0	12		1		292/275	17.1/5.4	8		1	
	III–IV	187/175	27.8/5.5	10	0.039	1.202 (0.993–1.456)	0.059	185/178	13.5/3.7	8	0.119	1.144 (0.947–1.381)	0.163
Extent of resection	STR or NTR	153/150	19.6/2.0	12		1		150/147	2.0/2.0	8		1	
	GTR	328/288	36.3/10.2	11	0.000	0.667 (0.545–0.817)	0.000	327/306	22.0/6.0	8	0.000	0.635 (0.519–0.777)	0.000
Radiotherapy	No	44/37	40.9/15.9	8		1		41/41	17.1/2.4	10		1	
	CRT	122/104	21.3/12.9	9		1.089 (0.747–1.589)	0.657	121/107	15.7/11.4	7		1.195 (0.830–1.720)	0.338
	GK	315/297	33.3/5.3	11	0.846	1.096 (0.778–1.544)	0.599	315/305	15.6/2.6	8	0.543	1.108 (0.798–1.537)	0.54
Chemotherapy	No	283/270	24.7/0.0	9		1		282/282	14.9/0.0	7		1	
	Yes	198/168	39.9/14.2	12	0.000	0.643 (0.528–0.782)	0.000	195/171	16.9/11.6	8	0.000	0.731 (0.602–0.889)	0.002

OS: Overall survival; PFS: Progression free survival; WHO: World Health Organization; GTR: Gross-total resection; NTR: Near-total resection;

STR: Sub-total resection; CRT: Conformal radiation therapy; GK: Gamma knife; SR: Survival rate; MST: Median survival time(months).

HR: Hazard ratio; 95% CI: 95% Confidence interval.

Log-rank p values were calculated from Chi-Square test.

*p* values were calculated from Wald test.

*p* < 0.05 indicates statistical significance.

**Table 3:** Univariate analysis of the impact of clinical factors on low-grade glioma patient OS and PFS

Variable	Classification	No. of patients/events	OS					No. of patients/events	PFS				
			1/3-(year) SR (%)	MST	Log-rank *p*	HR (95% CI)	*p*		1/3-(year) SR (%)	MST	Log-rank *p*	HR (95% CI)	*p*
Gender	Male	157/139	35.0/9.8	12		1		156/143	21.8/7.9	8		1	
	Female	137/124	30.7/8.4	12	0.241	1.142 (0.895–1.456)	0.285	136/132	11.8/2.5	8	0.097	1.197 (0.944–1.518)	0.137
Age (years)	< 40	144/127	33.3/10.4	12		1		142/131	17.6/6.7	8		1	
	≥ 40	150/136	32.7/7.7	11	0.487	1.082 (0.849–1.378)	0.526	150/144	16.7/3.9	8	0.325	1.113 (0.878–1.411)	0.377
WHO grade	I	35/30	31.4/12.0	9		1		35/34	20.0/2.9	6		1	
	II	259/233	33.2/8.7	12	0.792	0.954 (0.652–1.396)	0.810	257/241	16.7/5.8	8	0.413	0.847 (0.610–1.252)	0.462
Extent of resection	STR or NTR	97/95	21.6/2.1	12		1		95/93	2.1/2.1	8		1	
	GTR	197/168	38.6/12.5	11	0.001	0.677 (0.522–0.878)	0.003	197/182	24.4/7.1	8	0.000	0.642 (0.496–0.831)	0.001
Radiotherapy	No	24/19	45.8/20.8	12		1		22/22	22.7/0.0	10		1	
	CRT	73/62	20.5/13.2	12		1.270 (0.758–2.128)	0.363	73/65	13.7/10.8	8		1.373 (0.843–2.237)	0.202
	GK	197/182	36.0/6.8	12	0.605	1.196 (0.745–1.920)	0.459	197/188	17.8/3.8	8	0.299	1.176 (0.754–1.832)	0.474
Chemotherapy	No	170/160	28.2/0.0	10		1		170/170	16.5/0.0	8		1	
	Yes	124/103	39.5/16.2	12	0.000	0.665 (0.517–0.855)	0.001	122/105	18.0/13.3	8	0.004	0.722 (0.563–0.926)	0.010

OS: Overall survival; PFS: Progression free survival; WHO: World Health Organization; GTR: Gross-total resection; NTR: Near-total resection.

STR: Sub-total resection; CRT: Conformal radiation therapy; GK: Gamma knife; SR: Survival rate; MST: Median survival time (months).

HR: Hazard ratio; 95% CI: 95% Confidence interval.

Log-rank p values were calculated from Chi-Square test.

*p* values were calculated from Wald test.

*p* < 0.05 indicates statistical significance.

**Table 4:** Univariate analysis of the impact of clinical factors on high-grade glioma patient OS and PFS

Variable	Classification	No. of patients/events	OS					No. of patients/events	PFS				
			1/3-(year) SR (%)	MST	Log-rank *p*	HR (95% CI)	*p*		1/3-(year) SR (%)	MST	Log-rank *p*	HR (95% CI)	*p*
Gender	Male	107/102	23.4/2.3	11		1		106/105	11.3/0.9	8		1	
	Female	80/73	33.7/8.4	9	0.586	0.926(0.684-1.254)	0.619	79/73	16.5/7.4	7	0.281	0.863(0.639-1.167)	0.340
Age(years)	< 40	64/59	32.8/6.6	10		1		63/60	15.9/4.8	8		1	
	≥ 40	123/116	25.2/5.0	10	0.418	1.126(0.822-1.541)	0.460	122/118	12.3/3.1	7	0.388	1.129(0.827-1.542)	0.445
WHO grade	III	124/116	29.8/5.1	11		1		123/118	13.8/4.1	8		1	
	IV	63/59	23.8/6.3	9	0.471	1.111(0.812-1.521)	0.510	62/60	12.9/3.2	7	0.443	1.114(0.816-1.521)	0.496
Extent of resection	STR or NTR	56/55	16.1/1.8	9		1		55/54	1.8/1.8	7		1	
	GTR	131/120	32.8/6.9	10	0.002	0.624(0.451-0.864)	0.004	130/124	18.5/4.5	8	0.001	0.607(0.439-0.840)	0.003
Radiotherapy	No	20/18	35.0/10.0	8		1		19/19	10.5/0.0	6		1	
	CRT	49/42	22.4/12.6	9		0.894(0.510-1.566)	0.695	48/42	18.8/12.5	7		1.005(0.579-1.744)	0.987
	GK	118/115	28.8/2.5	10	0.775	1.008(0.612-1.660)	0.976	118/117	11.9/0.8	8	0.939	1.055(0.648-1.720)	0.829
Chemotherapy	No	113/110	19.5/1.8	9		1		112/112	12.5/3.6	7		1	
	Yes	74/65	40.5/11.3	12	0.001	0.617(0.450-0.846)	0.003	73/66	15.1/9.4	8	0.054	0.762(0.557-1.042)	0.088

OS: Overall survival; PFS: Progression free survival; WHO: World Health Organization; GTR: Gross-total resection; NTR: Near-total resection.

STR: Sub-total resection; CRT: Conformal radiation therapy; GK: Gamma knife; SR: Survival rate; MST: Median survival time (months).

HR: Hazard ratio; 95% CI: 95% Confidence interval.

Log-rank *p* values were calculated from Chi-Square test.

*p* values were calculated from Wald test.

*p* < 0.05 indicates statistical significance.

According to Log-rank tests and Cox regression analysis, the two SNPs we studied were not correlated with OS or PFS in glioma patients (Table 5). However, we found that the *IL-10* rs1800871 C/T genotype was associated with increased OS in low-grade glioma cases (3-year survival rate = 13.1%, median survival = 12 months, Log-rank *p* = 0.019, HR=0.745, 95% CI=0.573–0.969, *p* = 0.028), but not high-grade cases. We also found that the *PRKDC* rs7003908 C/C genotype correlated with poor prognosis in high-grade glioma patients as measured by OS (3-year survival rate = 0.0%, median survival = 6 months, Log-rank *p* = 0.000, HR = 4.556, 95% CI = 1.936–10.721, *p* = 0.001) and PFS (3-year survival rate = 0.0%, median survival = 4 months, Log-rank *p* = 0.000, HR = 4.430, 95% CI = 1.884–10.416, *p* = 0.001), although this was not observed in low-grade glioma patients.

**Table 5:** Univariate analysis of the association between rs1800871, rs7003908 and glioma patient OS and PFS

SNP-ID	Genotype	No. of patients/events	OS					No. of patients/events	PFS				
			1/3-(year) SR (%)	MST	Log-rank *p*	HR (95% CI)	*p*		1/3-(year) SR (%)	MST	Log-rank *p*	HR (95% CI)	*p*
low-grade I–II												
rs1800871	C/C	36/35	25.0/5.6	10		1		36/35	13.9/2.8	7		1	
	C/T	129/109	39.5/13.1	12		0.745 (0.573–0.969)	0.028	129/118	23.3/8.1	8		0.799 (0.619–1.033)	0.087
	T/T	126/116	28.6/5.3	11	0.019	1.098 (0.752–1.603)	0.627	124/119	12.1/3.5	8	0.079	1.087 (0.745–1.585)	0.665
rs7003908	A/A	188/168	34.0/8.7	12		1		186/176	17.2/5.2	8		1	
	C/A	86/76	32.6/11.4	12		0.965 (0.736–1.265)	0.796	86/80	18.6/5.8	8		0.914 (0.701–1.190)	0.503
	C/C	20/19	25.0/0.0	12	0.843	1.105 (0.687–1.777)	0.68	20/19	10.0/5.0	9	0.755	0.956 (0.595–1.536)	0.852
high-grade III–IV												
rs1800871	C/C	22/20	31.8/9.1	11		1		22/20	18.2/9.1	8		1	
	C/T	93/88	24.7/4.3	9		1.133 (0.824–1.557)	0.444	91/89	13.2/2.2	7		1.105 (0.806–1.516)	0.536
	T/T	71/67	29.6/4.9	10	0.467	0.883 (0.536–1.455)	0.626	71/68	11.3/4.2	8	0.598	0.908 (0.551–1.496)	0.704
rs7003908	A/A	112/106	32.1/3.1	10		1		110/107	13.6/2.7	8		1	
	C/A	69/63	23.2/8.7	10		0.935 (0.684–1.279)	0.676	69/65	14.5/5.8	8		0.979 (0.718–1.333)	0.891
	C/C	6/6	0.0/0.0	6	0.000	4.556 (1.936–10.721)	0.001	6/6	0.0/0.0	4	0.000	4.430 (1.884–10.416)	0.001

SR: Survival rate; MST: Median survival time (months).

HR: Hazard ratio; 95% CI: 95% Confidence interval.

Log-rank *p* values were calculated using the Chi-Square test.

*p* values were calculated using the Wald test.

*p* < 0.05 indicates statistical significance.

### Multivariate analysis

Multivariate Cox regression analysis demonstrated that SNP genotype was an independent prognostic factor for OS and PFS after adjustment for the various clinical factors. In the present study, we identified correlations between rs1800871, rs7003908 and prognosis in different glioma grades (Table 6). The *IL-10* rs1800871 C/T genotype correlated with better OS in low-grade glioma patients (adjusted HR = 0.736, 95% CI = 0.565–0.958, *p* = 0.022) and the *PRKDC* rs7003908 C/C genotype correlated with worse OS (adjusted HR = 4.288, 95% CI = 1.808–10.169, *p* = 0.001) and PFS (adjusted HR = 3.783, 95% CI = 1.601–8.943, *p* = 0.002) in high-grade glioma patients.

**Table 6:** Multivariate analysis of the association between rs1800871, rs7003908 and glioma patient OS and PFS

SNP-ID	Genotype	OS		PFS	
		Adjusted HR (95% CI)	*p*	Adjusted HR (95% CI)	*p*
low-grade I–II					
rs1800871	C/C	1		1	
	C/T	0.736 (0.565–0.958)	0.022	0.789 (0.610–1.020)	0.071
	T/T	1.073 (0.735–1.567)	0.716	1.059 (0.726–1.545)	0.767
rs7003908	A/A	1		1	
	C/A	0.968 (0.738–1.270)	0.817	0.918 (0.705–1.196)	0.527
	C/C	1.119 (0.696–1.799)	0.643	0.962 (0.599–1.547)	0.874
high–grade III–IV					
rs1800871	C/C	1		1	
	C/T	1.149 (0.832–1.588)	0.399	1.169 (0.850–1.659)	0.336
	T/T	0.879 (0.533–1.449)	0.612	0.929 (0.564–1.532)	0.774
rs7003908	A/A	1		1	
	C/A	1.038 (0.755–1.427)	0.82	1.033 (0.756–1.412)	0.839
	C/C	4.288 (1.808–10.169)	0.001	3.783 (1.601–8.943)	0.002

OS: Overall survival; PFS: Progression free survival.

HR: Hazard ratio; 95% CI: 95% Confidence interval.

*p* values were calculated using the Wald test.

*p* < 0.05 indicates statistical significance.

## DISCUSSION

Gliomas are the most common primary tumors of the central nervous system and several patient prognosis predictors have been identified, including patient age, WHO tumor grade, extent of tumor resection, and radiotherapy and chemotherapy regimen(s) [15]. Consistent with previous studies [16, 17], we found that chemotherapy and the extent of resection were key prognostic factors in glioma patients. Prognoses differed between low-grade and high-grade glioma patients. After WHO grade stratification, we found that rs1800871 in *IL-10* correlated with increased OS in low-grade glioma cases, while rs7003908 in *PRKDC* correlated with poor prognosis in high-grade cases.

Ours was the first study to associate the *IL-10* rs1800871 C/T genotype with improved survival in low-grade glioma patients. *IL-10* gene polymorphisms have been associated with multiple cancers, such as gastric [18], breast [19], and non-small cell lung cancer [20], as well as gliomas [9, 21]. Inflammation plays roles in all phases of tumor development, and IL-10, a well-known immuno-modulatory cytokine, may provide a functional link between inflammation and cancer [22]. Acuner-Ozbabacan, *et al.* [22] found that IL-10 deficiency allowed for pro-inflammatory cytokine induction and hindered anti-tumor immunity, thereby promoting tumor growth. Tanikawa, *et al.* [23] observed that high serum IL-10 levels enhanced the tumor-specific immune response and reduced tumor growth. These results were inconsistent with earlier findings by Kim, *et al.* [24], who proposed that IL-10 inhibition would promote the anti-tumor immune response. These contrary findings may be due to the intrinsically pleiotropic biological activity of IL-10 and the variability of cancer models [25], or possibly genetic factors between different races. The exact function of IL-10 in glioma is not clear; further studies are required to elucidate the mechanisms underlying the association between *IL-10* polymorphisms and glioma patient prognosis.

This study was also the first to demonstrate that the *PRKDC* rs7003908 C/C genotype was correlated with poor prognosis in high-grade glioma patients. Rs7003908 was previously associated with increased risk of glioblastoma multiforme [14], hepatocellular carcinoma [26], prostate cancer [27], and bladder cancer [28]. *PRKDC* encodes the catalytic subunit of DNA-dependent protein kinase (DNA-PKcs), which is critical in the DNA damage response (DDR) and maintenance of genomic stability [29]. Goodwin, *et al.* [30] suggested that DNA-PKcs suppression inhibited tumor metastasis. Their group observed that DNA-PKcs induced cell migration, invasion, and metastasis, and was highly activated in advanced tumors, independent of DNA damage indicators. The same group [29] showed that *PRKDC*, as a transcriptional regulator, might promote tumor initiation and progression. Additionally, *PRKDC* expression and activity is increased in numerous tumor types, such as colorectal cancer [31], esophageal cancer [10], and B-cell chronic lymphocytic leukemias [11]. In the present study, we observed reduced OS and PFS in high-grade glioma patients carrying the *PRKDC* rs7003908 C/C genotype. This mutation may be associated with increased *PRKDC* expression, activity, or receptor activation, any of which might promote cell proliferation.

In conclusion, our data indicated that chemotherapy and extent of tumor resection were associated with improved glioma patient prognosis. IL-10 SNP rs1800871 was associated with improved survival in low-grade glioma cases, while *PRKDC* SNP rs7003908 was correlated with poor prognosis in high-grade patients. Although the exact mechanisms remain unclear, our results suggest that these two SNPs may be useful biomarkers for predicting glioma patient outcomes. Additionally, IL-10 and DNA-PKcs may be promising therapeutic targets for treatment of advanced malignancies. Further functional studies are needed to evaluate associations between rs1800871 and rs7003908 polymorphisms and glioma patient prognosis.

## MATERIALS AND METHODS

### Subjects

A total of 481 patients diagnosed with glioma at the Department of Neurosurgery, Tangdu Hospital, Fourth Military Medical University, Shaanxi Province (Xi'an, China) between September 2010 and April 2016 were randomly enrolled in this study. We used the following detailed selection criteria: recently diagnosed and histologically confirmed to have glioma (glioma was diagnosed via imaging and pathological analysis); Han Chinese patient with no kinship; underwent regular follow-up; peripheral blood samples available; no previous history of other cancers; and no prior treatment for glioma or prior treatment with chemotherapy or radiotherapy. Histopathological diagnosis was confirmed according to the World Health Organization (WHO) classification in 2007.

Our study protocol adhered to the principles of the Declaration of Helsinki and was reviewed and approved by the Ethics Committee of Tangdu Hospital. All patients gave written informed consent prior to participation in the study.

### DNA extraction and genotyping

Genomic DNA was extracted from glioma patient peripheral blood samples (5 mL) using GoldMag-Mini Whole Blood Genomic DNA Purification Kits (GoldMag. Co. Ltd., Xi'an, China) according to the manufacturer's protocols. DNA quantity was evaluated by spectrophotometry (DU530UV/VIS spectrophotometer, Beckman Instruments, Fullerton, CA, USA). We designed polymerase chain reaction (PCR) and extension primers for the SNPs using the Sequenom MassARRAY Assay Design 3.0 software (Sequenom, San Diego, CA, USA). Sequenom MassARRAY RS1000 was utilized to genotype SNPs according to the manufacturer's protocol. We used Sequenom Typer 4.0 software for data management and analysis.

### Clinical data and follow-up

Patient treatment and survival (overall and progression-free) information was collected from a retrospective review of patient medical records or consultation with treating physicians. A standardized questionnaire was used to collect clinical data, including the date of diagnosis, follow-up date(s), patient age and gender, tumor histologic type, extent of resection, exact pathology, WHO grade, surgical methods, and postoperative radiotherapy and chemotherapy regimen(s). Follow-up consisted of outpatient visits, telephone interviews, and written communication with patients or their families. Clinical follow-up was performed in single-blind fashion with death as the end point. Glioma patients who were alive at the end of the follow-up period were excluded from the study. Data were stored electronically using the EpiData3.02 software, and validation, revision and conversion of assigned values were performed to establish the glioma patient database used for analysis.

### Statistical analyses

SPSS 17.0 (SPSS, Chicago, IL, USA) was used to analyze follow-up and experimental data. Log-rank tests were used to compare survival curves. Univariate and multivariable Cox proportional hazard models were used to calculate crude and adjusted hazard ratios (HRs) and 95% confidence intervals (CIs), respectively. Two-sided *p*-values < 0.05 were considered statistically significant and were calculated using the Wald test. HRs were adjusted for other factors that could affect glioma outcome, such as patient age and gender, and extent of tumor resection.
